# The Absolute Number of Oligodendrocytes in the Adult Mouse Brain

**DOI:** 10.3389/fnana.2018.00090

**Published:** 2018-10-30

**Authors:** Bruna Valério-Gomes, Daniel M. Guimarães, Diego Szczupak, Roberto Lent

**Affiliations:** ^1^Institute of Biomedical Sciences, Federal University of Rio de Janeiro, Rio de Janeiro, Brazil; ^2^D’Or Institute for Research and Education, Rio de Janeiro, Brazil

**Keywords:** oligodendroglia, isotropic fractionator, Olig2, brain cell number, cellularity

## Abstract

The central nervous system is a highly complex network composed of various cell types, each one with different subpopulations. Each cell type has distinct roles for the functional operation of circuits, and ultimately, for brain physiology in general. Since the absolute number of each cell type is considered a proxy of its functional complexity, one approach to better understand how the brain works is to unravel its absolute cellularity and the quantitative relations between cell populations; in other words, how one population of cells is quantitatively structured, in relation to another. Oligodendrocytes are one of these cell types – mainly, they provide electric insulation to axons, optimizing action potential conduction. Their function has recently been revisited and their role extended, one example being their capability of providing trophic support to long axons. To determine the absolute cellularity of oligodendroglia, we have developed a protocol of oligodendrocyte quantification using the isotropic fractionator with a pan-marker for this cell type. We report a detailed assessment of specificity and universality of the oligodendrocyte transcription factor 2 (Olig2), through systematic confocal analyses of the C57BL/6 mouse brain. In addition, we have determined the absolute number (17.4 million) and proportion (about 20%) of this cell type in the brain (and in different brain regions), and tested if this population, at the intraspecific level, scales with the number of neurons in an allometric-based approach. Considering these numbers, oligodendrocytes proved to be the most numerous of glial cells in the mouse brain.

## Introduction

The central nervous system (CNS) is a highly complex structure, mostly composed of two large cell populations: neurons and glial cells. The classical notion that glial cells’ only function is to provide support for neurons, acting as secondary elements in brain physiology, has been overridden by an abundant set of evidence showing that cells such as astrocytes and oligodendrocytes are paramount for the information processing by neuronal circuitry (Takahashi et al., 2011; Edgar and Sibille, 2012; von Büdingen et al., 2015). In particular, the roles of oligodendrocytes and their precursors have been rediscovered, beyond their ability to insulate the axon. It has been shown that they provide trophic support for long axons (Wilkins et al., 2001; Nave, 2010), signals for white matter angiogenesis (Yuen et al., 2014), and support for the blood-brain barrier, by increasing its tightness (Seo et al., 2014). The differentiation of oligodendrocyte progenitors into mature cells influences white matter plasticity (Boulanger and Messier, 2014), and has been attributed a fundamental role in regulating complex cognitive behaviors, such as social interaction (Makinodan et al., 2012). In addition, oligodendrocyte dysfunction has been considered pivotal in many neurological and psychiatric disorders, such as multiple sclerosis (Vassall et al., 2015) and schizophrenia (Windrem et al., 2017). Evidence, therefore, suggests that oligodendrocytes are paramount for the maintenance of brain function, and may become potential targets for therapeutical approaches under pathological conditions.

Knowing the absolute quantitative cell composition of the brain is important as a normative frame of reference for pathological conditions, for age-dependent changes and for the effect of external, environmental interventions. In addition, it is useful to understand the quantitative balance among different cell types, and, ultimately, which logistics development has followed to build the brain. It has been shown that the number of non-neuronal cells, mostly glial cells, is a good predictor of the number of neurons, even in an intraspecific scale (Herculano-Houzel et al., 2015). This is interesting because not every allometric relation established among individuals of different species applies to the intraspecific level, as happens with body or brain masses and number or density of neurons, which strongly correlate across mammalian species (Armstrong, 1990). So, the quantitative relations between glial cells and neurons seem fundamental to unravel scaling rules both at the intra- and interspecific levels. Regarding a possible relation between glial cells and neurons, a question arises: if the final amount of cell types in the brain is determined by subtle regulation of precursor proliferation and cell death during development, it could be that glial cells and neurons communicate along the developmental time line, in such a way that final absolute numbers become correlated. This question may be tackled at a general level (glial and neuronal cells), at a more specific level (oligodendrocytes and neurons, for instance) or with an even more specific approach (the oligodendrocytes ensheathing retinal ganglion cells, for example). We here examined this issue by comparing general numbers of oligodendrocytes with those of neurons in specific regions of the brain.

To achieve this aim it is necessary, through systematic confocal analyses, to assess the labeling specificity (i.e., the marker labels only one particular cell type) and universality (i.e., it labels all cells of that type) of the oligodendrocyte transcription factor 2 (Olig2), a basic helix-loop-helix transcription factor, using an optimized methodology similar to the one proposed by Chen et al. (2015). With this marker, we have determined the absolute number and proportion of cells of the oligodendroglial lineage, and then investigated the allometric relation between oligodendrocytes and neurons, in different regions of the murine brain. We have employed the isotropic fractionator (Herculano-Houzel and Lent, 2005; Azevedo et al., 2009; Andrade-Moraes et al., 2013), which was recently validated for neuronal and non-neuronal quantification (Bahney and von Bartheld, 2014, 2018; Miller et al., 2014; Ngwenya et al., 2017), to count Olig2+ nuclei in mice. This method allows turning highly anisotropic neural structures of complex architectonic organization into homogeneous nuclei suspensions, which can be immunostained and quantified under fluorescence microscopy. The technique has been recently optimized in different ways: the homogenization procedure was automated (Azevedo et al., 2013) and counting by use of a flow cytometer has been proven viable (Collins et al., 2010; Young et al., 2012).

We here report the absolute number of oligodendroglial cells in different regions of interest in the adult mouse brain, and provide evidence that oligodendrocytes are likely to be the most abundant glial cell within most regions of the murine adult brain. In addition, by estimating the composition of neurons, oligodendrocytes and other cells at an intraspecies level, we show that the number of oligodendrocytes does not necessarily correlate with the number of neurons in the brain and dissectible brain regions, as reported for an interspecies level of comparison.

## Materials and Methods

### Animals

All animal protocols were approved with the terms of the Ethics Commission on Animal Use (Protocol # 01200.001568/2013-87) of the Federal University of Rio de Janeiro. We have used 14 C57BL/6 mice (11 for nuclei quantification and allometric correlations, and 3 for immunohistochemical validation of the Olig2 marker). All animals were males and adults (P90–P100). The sample size was estimated by statistical calculation (see the Statistical Approach section below).

Animals were housed under standard conditions of temperature and humidity, with an automatically controlled day/night cycle of 12 h, and *ad libitum* access to food and water. Mice were weaned at the age of P21 and were kept with other animals of the same sex (four mice per cage) until the day of the experiment-guided euthanasia. This was achieved on the day of the experiments by an intraperitoneal injection of ketamine (100 mg/kg) and xylazine (10 mg/kg) followed by transcardiac perfusion and fixation.

### Brain Fixation and Removal

After full sedation, animals were transcardially perfused with saline solution (0.9% sodium chloride), followed by fixation with 4% phosphate-buffered paraformaldehyde.

After fixation, animals were decapitated and craniotomy followed. The first vertebra was considered the caudal limit of the medulla. The optic chiasm was carefully excised and since the cerebellar paraflocculus was often damaged during dissection, we removed it from all brains. Brains and regions were weighed immediately after dissection to avoid dehydration.

### Dissection of Regions of Interest (ROIs)

**FIGURE 1:**
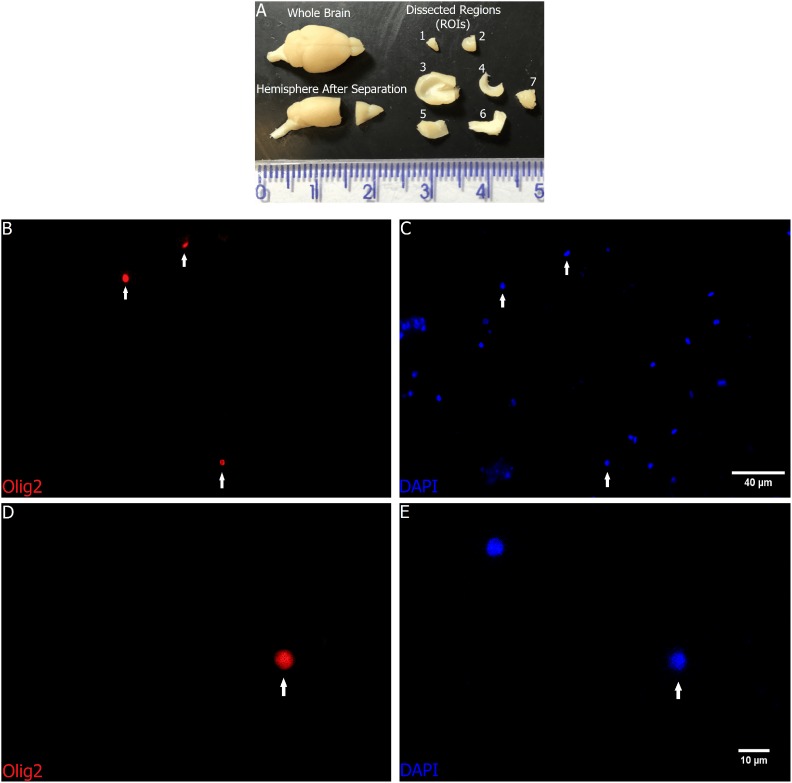
Regions of interest before and after fractionation. All dissected ROIs in **(A)**: Upper left – Whole brain; Lower left – A hemisphere after separation of *anterior cortex plus olfactory bulb* from the rest of the brain; Upper and lower right – 1: *olfactory bulb*, 2: *anterior neocortex*, 3: *remaining cortex* (including the piriform cortex), 4: *hippocampus*, 5: *diencephalon plus basal ganglia*, 6: *brainstem*, and 7: *cerebellum*. Scale in centimeters. **(B–E)** Representative images from distinct fields with **(B,C)** lower magnification, and **(D,E)** higher magnification of the cortex nuclei suspension after fractionation, with staining and immunocytochemistry to Olig2 **(B,D)** and DAPI **(C,E)**. The images show nuclei doubly stained with DAPI and Olig2 (arrows). Scale bars: 40 μm **(B,C)** and 10 μm **(D,E)**.

After removing the brain, the following ROIs were dissected: *olfactory bulb* (plus tract), *anterior neocortex*, *remaining cortex* (including the piriform cortex), *hippocampus*, *diencephalon plus basal ganglia*, *brainstem*, and *cerebellum* (Figure 1A).

The first step was to divide the brain sagittally with a razor blade. The next steps were performed for both hemispheres separately. Using a mold with the midplane of the hemisphere facing down, the surface of the cortex aligned to a horizontal line, and the rostral pole of the callosal genu aligned to a vertical line, a coronal cut was made to separate what we call *anterior cortex plus olfactory bulb* from the rest of the encephalon.

With the medial aspect of the anterior cortex (plus olfactory bulb) facing down, a small incision was made with a scalpel, separating the olfactory bulb and its tract from the rest. Following the rhinal fissure, the piriform cortex was separated from the anterior cortex and added to the posterior cortex, both then composing the ROI named *remaining cortex*. Any residual gray matter below the anterior neocortex was dissected out and added to the diencephalon plus basal ganglia. The posterior cortex was removed by small incisions under the corpus callosum and cortical white matter and separated from the hippocampal formation with a small surgical spatula positioned between the hippocampus and the lateral ventricle.

Finally, with the medial aspect of the remaining structures still facing down, a coronal cut was made rostral to the superior colliculi, thus separating the whole brainstem from the more anterior structures.

### Immunohistochemistry and Confocal Imaging

One of the objectives of this work was to estimate the absolute number of oligodendroglial cells in the mouse brain. Despite the existence of some good markers of oligodendrocytes commonly used in immunocytochemical approaches, most of them are cytoplasmic and therefore unsuitable for counting nuclei using the isotropic fractionator. The first need, thus, is to choose a reliable nuclear marker for estimating the absolute composition of oligodendrocytes and assess its specificity and universality for this particular brain cell type. For this purpose, one needs firstly to co-label the chosen nuclear marker of oligodendrocytes with cytoplasmic markers of different cell types, to establish that it only colocalizes with oligodendrocyte cytoplasmic markers (specificity), and that it does so for all oligodendrocytes in the brain (universality). The chosen nuclear marker for oligodendrocytes was Olig2, well known to label the nucleus of this particular glial cell type (Yokoo et al., 2004).

The cytoplasmic markers chosen for oligodendrocytes were: chondroitin sulfate proteoglycan 4 (NG2 or CSPG4) (Nishiyama et al., 1996) for oligodendrocyte-precursor cells (OPCs); 2′,3′-Cyclic-nucleotide 3′-phosphodiesterase (CNPase) for pre- and myelinating oligodendrocytes (Scherer et al., 1994), myelin basic protein (MBP); and adenomatous polyposis coli (APC) [aka CC1] (Bhat et al., 1996) for myelinating oligodendrocytes. For the other brain cell types, we chose hexaribonucleotide binding protein-3 (NeuN) (Mullen et al., 1992) for neurons; ionizing calcium-binding adaptor molecule 1 (IBA-1) for microglial cells, isolectin B4 (Ernst and Christie, 2006) and collagen IV for vascular and endothelial cells; and aldehyde dehydrogenase 1 family member L1 (ALDH1L-1) (Cahoy et al., 2008) as a universal and specific astrocyte marker, since glial fibrillary acidic protein (GFAP) labels processes of just one type of astrocyte.

After perfusion, the brains of three animals were submitted, for 4 days, to a cryopreservation protocol. On the 1st day, they were immersed in 4% phosphate-buffered paraformaldehyde at 4°C and in the three subsequent days, they were changed to 10, 20, and 30% sucrose solution in 0.1M PBS for 24 h each, at 4°C. Two brains were cleaved sagittally and both hemispheres were sliced parasagittally, while the third brain was sliced coronally. Samples were embedded in Optimal Cutting Temperature compound (OCT) (Sakura Tissue-Tek, 4583), frozen in liquid nitrogen for 5 min and stored at -80°C for 1–4 days before cryostat (Leica System CM 1860) sectioning at -20°C at 12 μm thickness. All slices were kept under -20°C until the immunostaining process. Proteins epitopes were recovered by immersion in citrate buffer solution (8.2% sodium citrate 0.1 M; 1.8% citric acid pH 6.1; 0.1% Tween 20) for 20 min at 96°C under water followed by 20 min at room temperature. After that, slices were washed with 0.1 M PBS for 10 min and then submitted for 2 h to a blocking-permeabilization solution (78% PBS with 0.1% Triton^TM^ X-100; 20% bovine serum albumin – from 5% aliquots in PBS 0.1 M (BSA) and 2% of normal goat serum (NGS).

For the immunolabeling, all the following primary antibodies were incubated in blocking solution (80% PBS with 0.1% Triton^TM^ X-100 and 20% BSA at 5%) in a wet chamber for 72 h at 4°C: anti-Olig2 mouse monoclonal IgG (1:50 MABN50; Millipore); anti-Olig2 rabbit monoclonal [EPR2673] IgG (1:50 AB109186; ABCAM); anti-NeuN mouse monoclonal [A60] IgG (1:50 MAB377; Millipore); anti-IBA-1 rabbit polyclonal IgG (1:100 SAJ2266; WAKO); anti-ALDH1L-1 rabbit polyclonal IgG (1:50 AB87117; ABCAM); anti-NG2 rabbit polyclonal IgG (1:50 AB5320; Millipore); anti-APC [CC-1] mouse monoclonal IgG (1:50 AB16794; ABCAM); anti-MBP rabbit polyclonal IgG (1:100 AB980; Millipore); anti-CNPase mouse monoclonal IgG (1:100 AB6319; ABCAM); anti-KI-67 rabbit polyclonal IgG (1:100 AB9269; Millipore); Biotinylated *Griffonia simplicifolia* Lectin I (GSL I) Isolectin B4 (1:200 B-1205; Vector Laboratories); anti-Collagen IV rabbit polyclonal IgG (1:200 AB6586; ABCAM). For the negative controls, samples were incubated for the same time period in blocking solution. After the primary incubation period, slices were washed thrice with PBS 0.1 M for 10 min and subsequently incubated with secondary antibodies in blocking solution (80% of PBS and 20% of BSA), including the negative control slices, for 2 h with gentle shaking at room temperature. The secondary antibodies used were Alexa 546 goat anti-mouse IgG (1:500 A11003; Invitrogen); Alexa 546 goat anti-rabbit IgG (1:500 AB60317; ABCAM); Alexa 488 goat anti-mouse IgG (1:500 AB150113; ABCAM); Alexa 488 goat anti-rabbit IgG (1:500 AP132JA4; Millipore) and Streptavidin Cy3 from *Streptomyces avidinii* (1:400 S6402; Sigma). After 2 h, the slices were washed thrice for 10 min in PBS 0.1 M and then stained with 1 mL of DAPI (20 mg/L D9542; Sigma) for 10 min with a last wash in PBS 0.1 M for 5 min. Finally, the slides were sealed with Fluoromount Aqueous Mounting Medium (F4680; Sigma).

All the image acquisitions were done using a Leica TCS-SPE and Zeiss Elyra PS.1 LSM 710 laser scanning confocal microscope at 10, 40, and 63× magnifications with Z-stacks (an average of 40 steps with 0.35 μm) and 1024 × 1024 resolution format. The acquired images were processed and analyzed by using ImageJ software (National Institutes of Health) and Adobe Photoshop CS2.

### Isotropic Fractionation

The protocol for fractionation was similar to the original method (Herculano-Houzel and Lent, 2005) reproduced by different laboratories (Collins et al., 2010; Rusznák et al., 2015; Gabi et al., 2016; Repetto et al., 2016), with some minor changes. Each region followed the same protocol, changing only the fractionation time, since different amounts of gray and white matters affect the mechanical resistance of the tissue to grinding. Each dissected region was fragmented and placed into the glass homogenizer, with a buffer-detergent solution (40 μM sodium citrate and 1% Triton^TM^ X-100), and through constant linear and rotatory movements of the pestle, the tissue was chemomechanically disrupted. Thus, with this technique, the highly anisotropic tissue was transformed into a suspension of intact nuclei. The duration of homogenization ranged from 5 min for mechanically softer regions (with more gray matter) such as the cerebellum, to 15 min for larger regions and those with more white matter such as the brainstem. The duration of homogenization for each region was chosen based on previous experiments – where the quality criteria were the absence of tissue clustering and disrupted nuclei.

After obtaining the isotropic suspension of nuclei, the material was transferred to a falcon tube. The homogenizer was rinsed with a solution of 0.1 M PBS to avoid losing nuclei, and added to the same falcon tube, where the volume was rounded up by adding PBS until reaching a convenient level for nuclei counting in a Neubauer chamber. Images were obtained from nuclei in the chamber using a Zeiss AxioPlan and Zeiss AxioImager with 20 and 40× objectives and processed using ImageJ and Adobe Photoshop CS2.

### Quantification of Nuclei

In order to count the absolute number of cells in the brain, we used the fluorescent nuclear stain DAPI (4′, 6-diamidino-2-phenylindole, 20 mg/L D9542; Sigma). This marker strongly binds to DNA regions rich in adenine and thymine, in nuclei of all cells. To each suspension of nuclei, we added 2% of DAPI (20 mg/L) and carefully agitated it for at least 30 times. At least four aliquots of 10 μL each were collected and placed in a Neubauer chamber for counting DAPI stained nuclei within some square sectors of the chamber. The number obtained was then multiplied by the volume in the falcon tube and by the factor 15,625 (factor related to the total number of squares considered for counting). By arithmetical extrapolation, we arrived at the number of total nuclei in the original suspension volume. Assuming that each cell in the nervous system has only one nucleus, the number of nuclei becomes an excellent proxy for the number of cells, and for this reason we will refer heretofore to numbers of cells as a synonym of number of nuclei.

For the Olig2+ nuclei quantification, from each fractionated ROI, one aliquot of 1000 μL was collected. The first step of the reaction was incubation for binding of the primary antibody. Aliquots were centrifuged and rinsed thrice in PBS 0.1 M (1500 rpm, 5 min each round of rinsing), supernatants were discarded, and pellets were resuspended. For the primary immunocytochemistry reaction, each sample was then incubated with 1 μL of BSA 5%, 15 μL of NGS, primary antibody anti-Olig2 1:50 and 35 μL of PBS 0.1 M. For better staining results, the primary incubation lasted 72 h.

For the secondary reaction after the primary incubation, samples were washed as reported before, then incubated with 2 μL of BSA 5%, 30 μL of NGS, 10 μL of DAPI (20 mg/L), secondary antibody Alexa 546 1:500 and 460 μL of PBS 0.1 M for 2 h.

After the incubation period, all aliquots were centrifuged and rinsed again, and at least 500 μL of PBS 0.1 M were added to the rinsed pellets. As in previous publications using the isotropic fractionator, the number of immunostained nuclei (Figures 1B,D) was determined out of at least 500 DAPI stained nuclei (Figures 1C,E). This is a relative counting, so in the end we have a proportion of Olig2+ nuclei. To arrive at the absolute number, we applied the obtained percentage to the total number of nuclei as estimated before.

To quantify neuronal nuclei, another aliquot of 1000 μL was collected from each processed region. Similar to the Olig2 staining procedure, samples were washed thrice and incubated for 24 h with 2 μL of BSA 5%, 30 μL of NGS primary antibody anti-NeuN 1:200 and 170 μL of PBS 0.1 M.

The reaction for binding of the secondary antibody Alexa 546 and the counting method of the NeuN+ nuclei were performed as described.

The number of the remaining cells (astrocytes + microglia + endothelial and ependymal cells) was obtained by subtracting the numbers of Olig2+ and NeuN+ cells from the total number of cells.

### Statistical Approach

First, a pilot study was designed with six animals, using all the techniques previously described to quantify the number of DAPI+, Olig2+, and NeuN+ nuclei. Using the standard deviation estimated within the sample, we calculated the sample size for a confidence level (CL) of at least 95%.

For the allometric analyses, the Spearman test was used to evaluate the magnitude of correlations. Sample size was estimated using the bivariate normal model of correlation for a *rho* as high as 0.75. Regression lines were not employed in graphs for the following reasons: (a) a correlation analysis was used, instead of a predictive model such as a linear regression (which would produce a regression line); and (b) the statistical design did not assume linearity or any preconceived relationship; thus, the data was modeled using Spearman’s correlation, that measures only the strength of the monotonic relation between variables (ranked analysis).

All statistical procedures were conducted using the softwares GraphPad Prism 6 and G^∗^Power 3.1.9.2 (Universität Düsseldorf). Data is shown in plot graphs with mean, standard error of the mean, coefficient of variation and, when a correlation proved significant, the *rho*.

Note that for some comparisons, the axes were cut down to improve data visualization.

## Results

### Olig2 Is a Universal (Pan) Marker

A universal or pan marker, by definition, must be present in all subtypes of a specific lineage, being thus capable of offering quantitative information about the whole lineage. Some studies demonstrate that there are three types of oligodendrocytes: those in the gray matter, those located in major tracts and commissures (interfascicular), and perivascular oligodendrocytes (Yokoo et al., 2004). To claim Olig2 as universal, thus, it should be demonstrated that this marker labels these three types.

**FIGURE 2:**
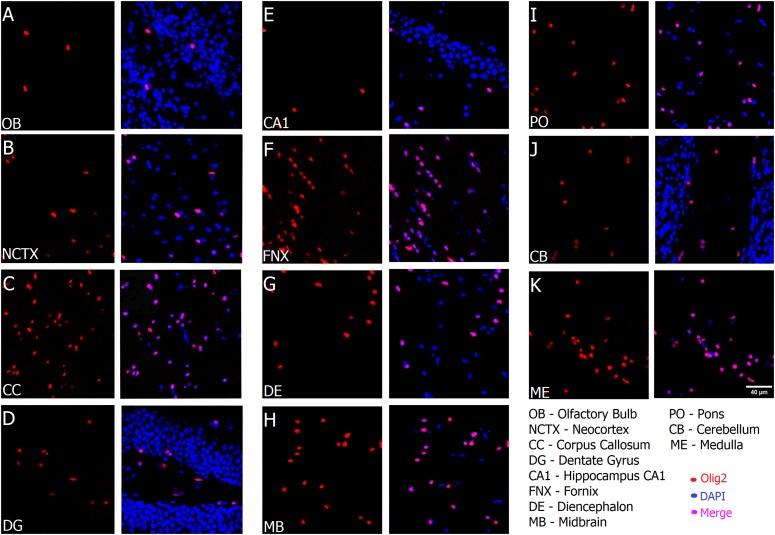
Oligodendrocytes are present in all regions of the mouse brain. From **(A–K)**, Olig2 (red) merged with DAPI (blue) and is detected in all anatomical compartments, including brain regions that originated from different embryonic subdivisions, and in both gray and white matter, in parasagittal frozen slices. Scale bar (same for all fields): 40 μm.

**FIGURE 3:**
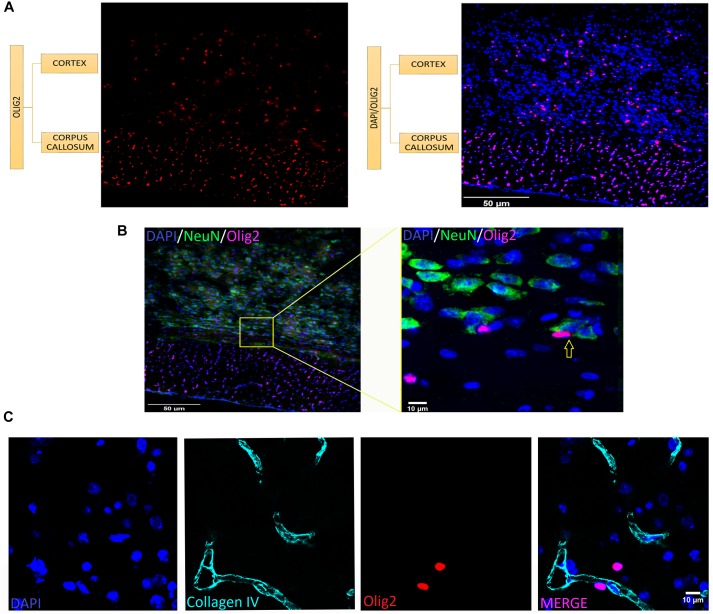
Oligodendrocytes are present at different densities and tissue locations in the brain. **(A)** Colocalization of Olig2 (red) and DAPI (blue) in gray (cerebral cortex) and white matter (corpus callosum). Notice higher density in the white matter. **(B)** Olig2+ cell (violet) in close apposition to NeuN+ cells (green) indicated in yellow arrow at a high magnification from a spread view, showing oligodendrocytes which are neuronal satellites. **(C)** Co-staining of vasculature basement membrane Collagen IV (cyan) and Olig2 (red) in the cerebral cortex showing perivascular oligodendrocytes (which are very close to blood vessels). Scale bars: 50 μm **(A,B)**, 10 μm **(B,C)**.

As shown in Figure 2, the typical small and round-to-oval nuclei of oligodendrocytes stand clearly after Olig2 labeling throughout both gray and white matter of the different ROIs with no exception, including anatomical compartments of distinct embryological origins, such as telencephalon, diencephalon, midbrain and hindbrain. However, since Olig2 is expressed early in development (Takebayashi et al., 2000; Ono et al., 2014), it is impossible to distinguish the subtypes of Olig2+ cells, unless by co-labeling them with a stage-specific marker. Lower magnification (Figure 3A) shows the broad distribution of Olig2+ cells, detected in different densities, lower in the gray matter (cerebral cortex), higher in the white matter (corpus callosum). In Figure 3B, an Olig2+ cell can be discerned in close apposition to a NeuN+ cell in the gray matter (yellow arrow), suggesting that the marker identifies perineuronal or satellite oligodendrocytes, a subtype known to embrace neuronal somata. Furthermore, perivascular oligodendrocytes were also labeled, as can be seen in Figure 3C, that shows a capillary immunostained with the vascular basement membrane Collagen IV marker in the cortex, together with some Olig2+ cells juxtaposed to it.

### Olig2 Is a Lineage Specific Marker

**FIGURE 4:**
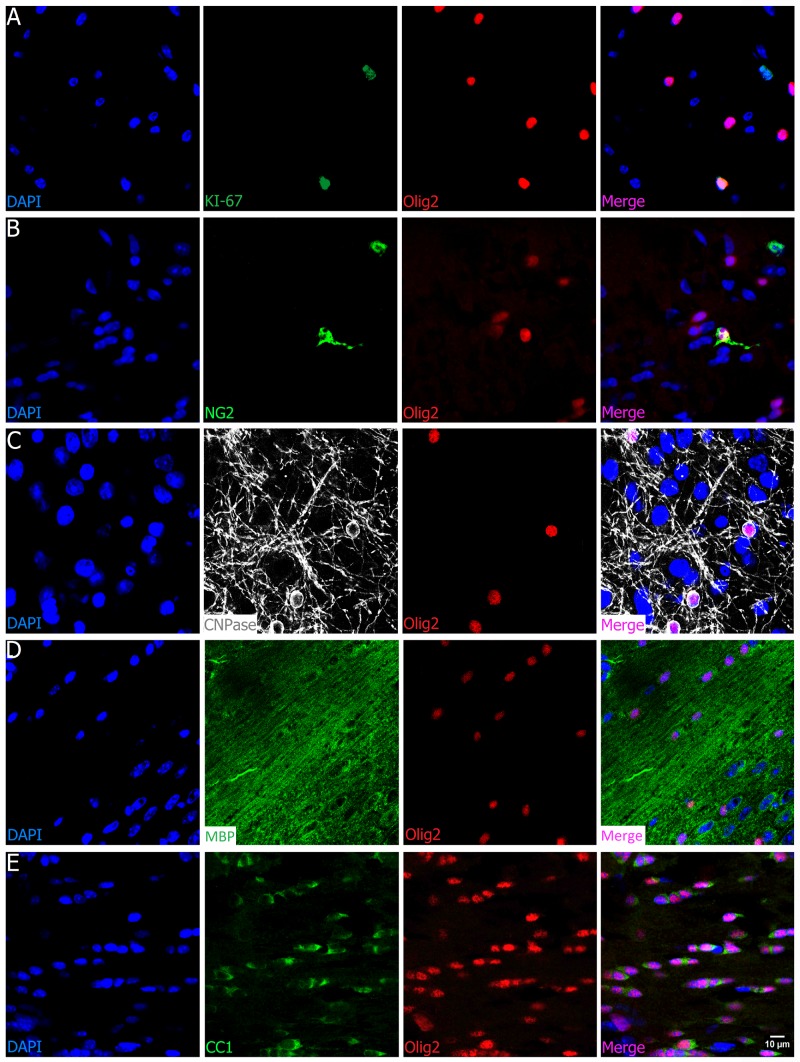
Olig2 is an oligodendrocyte universal marker. Olig2 (red) colocalizes (**A**, cortex and corpus callosum) with cell cycle KI-67 marker (green) as seen in the merge picture (orange), and also (**B**, cortex) with the chondroitin sulfate proteoglycan NG2 (green), evidence for its presence in the proliferative and migratory precursor population. (**C**, olfactory bulb) Olig2 is also present in the pre-myelinating and myelinating phase, since it colocalizes with CNPAse (gray), and (**D**, corpus callosum; **E**, fornix) with well-known adult oligodendrocytes markers such as MBP (green) and CC1, characteristic of the end of maturation. Scale bar (valid for all panels): 10 μm.

The oligodendrocyte transcription factor 2 (Olig2) is directly involved in differentiation and fate of oligodendrocytes and is present in the oligodendrocyte progenitor cells (OPC) as well as in mature (myelinating) ones. As shown in Figure 4A, Olig2 colocalizes with the cell cycle marker KI-67, although these cycling cells are not common to find and of course not all of them are oligodendrocyte precursors. Among these latter, Olig2 colocalizes with NG2+ cells (Figure 4B). This is evidence for its presence within the proliferative and migratory precursor cells population, known as NG2/PDGFRα (platelet-derived growth factor receptor alpha) precursor cells or polydendrocytes (OPC and late OPC). Besides, since Olig2 appears as early as the NG2/PDGFRα markers in OPCs, it is commonly easy to find a colocalization between Olig2 and NG2, despite a few NG2+ cells that do not colocalize with Olig2+ cells due to its expression in pericytes. In addition, Olig2 is also detected in non-myelinating and myelinating oligodendrocytes, as seen in examples of complete colocalization with CNPase+ cells (Figure 4C), in which expression starts at the pre-myelinating (intermediate) stage and continues until the myelinating (mature) stage. It also colocalizes with other well-established myelinating oligodendrocyte markers such as MBP (Figure 4D) and CC1 (Figure 4E).

**FIGURE 5:**
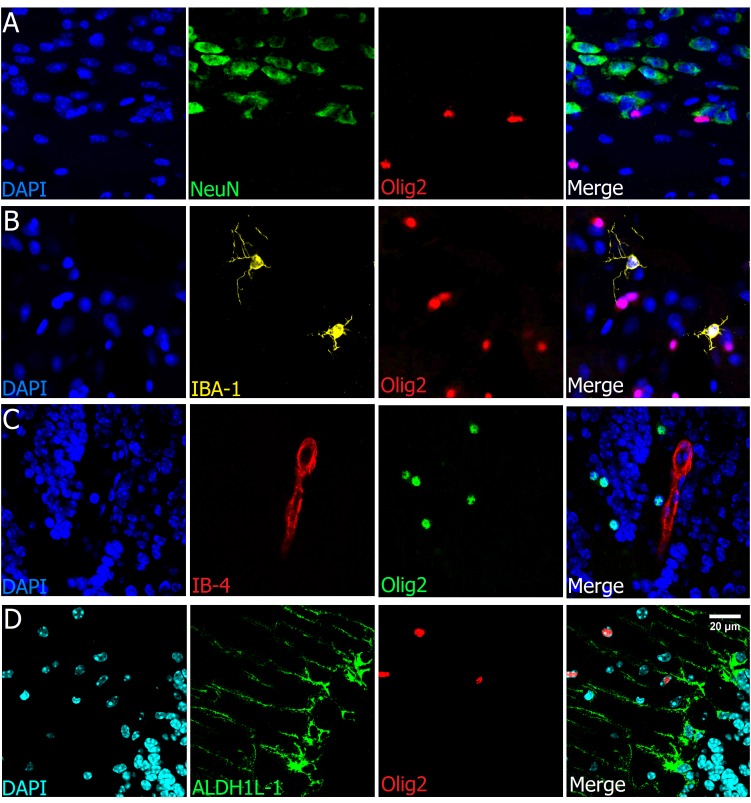
Olig2 is a lineage-specific marker of oligodendrocytes. Double labeling of Olig2 with other major cell types such as neurons (**A**, cortex), microglia (**B**, cortex), vasculature and endothelial cells (**C**, olfactory bulb), and astrocytes (**D**, cerebellum), showed no colocalization with those specific cell markers. Scale bar (valid for all photographs): 20 μm.

To assess that Olig2 is a lineage-specific marker, we tested its colocalization with neuronal, endothelial and glial nuclear/cytoplasmic markers. As shown in representative images (Figures 5A,B), there was neither colocalization of Olig2 with NeuN (neurons) nor with IBA-1, a well-known microglial marker. As there are some oligodendrocytes in close proximity to blood vessels and capillaries and as some have irregular-oval nuclear morphology, we needed to check whether the Olig2+ nuclei could colocalize with endothelial cell markers. However, although very close, they did not colocalize, as demonstrated by double staining with Olig2 and Isolectin-B4, a vasculature and endothelial cell marker (Figure 5C) and neither with Collagen IV vasculature basement membrane marker (Figure 3C). Concerning astrocytes, we tested the cytoplasmic marker ALDH1L-1, which labels not only processes but also the somata of all astrocytes (Cahoy et al., 2008), differently from GFAP, which labels processes of only one kind of astrocyte. As expected, we found no evidence of colocalization with nuclear Olig2+ cells (Figure 5D).

### Absolute Number of Oligodendrocytes in Comparison With Other Cells

Having established the adequacy of Olig2 as a specific and universal marker for oligodendrocytes, we moved to quantify the absolute numbers of these cells in the mouse brain.

All brains and ROIs were weighed for the purpose of allometric scaling analyses and possible detection of biases. As expected for standardized dissectible regions in an isogenic linage, the weight did not vary greatly (Table 1).

**Table 1:** Average weight of dissected structures and of the whole brain (both hemispheres), standard deviations (*SD*), and coefficients of variation (CV).

Structure	*N*	Average	*SD*	CV
		weight (g)		
Anterior neocortex	11	0.02264	0.003931	17.37%
Remaining cortex	11	0.1376	0.01875	13.62%
Olfactory bulb	11	0.02282	0.003842	16.84%
Hippocampus	11	0.03009	0.00359	11.93%
Diencephalon plus Basal Ganglia	11	0.07318	0.005759	7.87%
Brainstem	11	0.09082	0.00704	7.75%
Cerebellum	11	0.04827	0.00465	9.63%
Encephalon	11	0.4255	0.0342	8.04%


**FIGURE 6:**
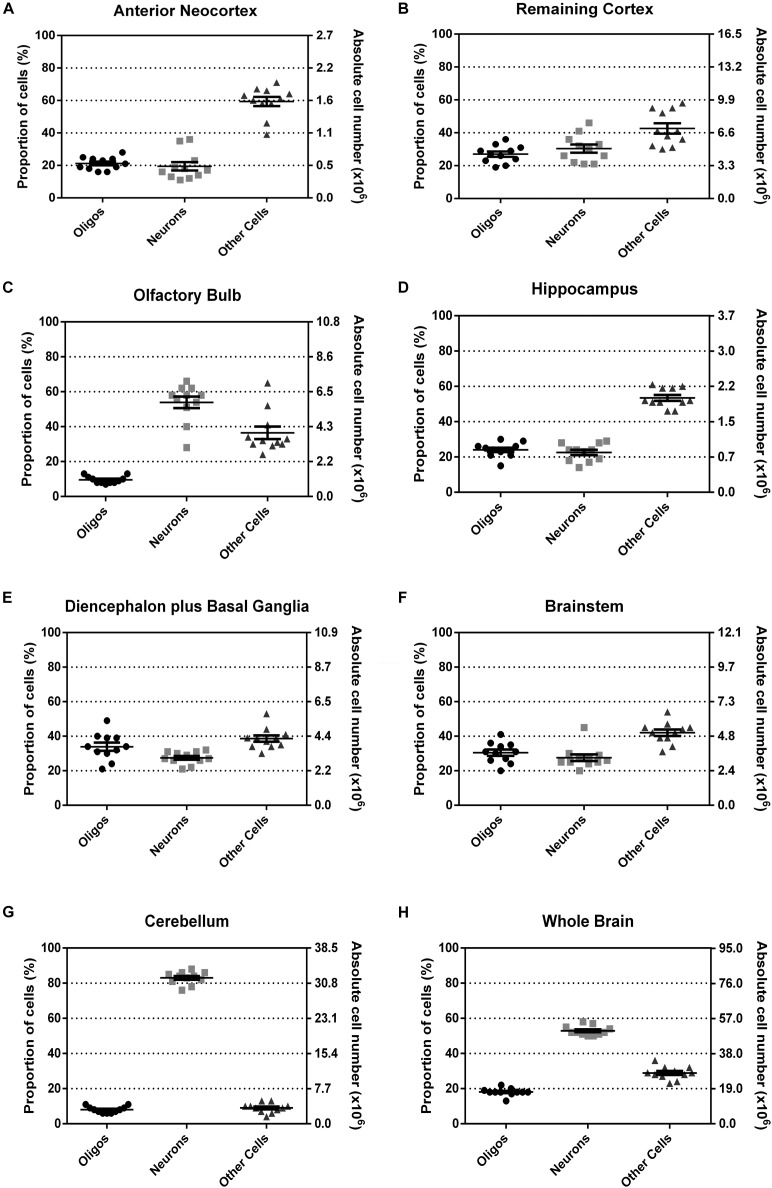
Relative and absolute number of oligodendrocytes, neurons and other cells, in percentage for all the dissected regions **(A–G)** and for the whole brain **(H)**. Data shown as scatter-plots, with mean and standard error of the mean, *n* = 11.

The proportion and absolute numbers of each cell type per ROI is summarized in Figure 6. The explicit statistical descriptive parameters can be found in Table 2. As can be seen by the tight distributions of cell numbers and proportions, the quantification of oligodendrocytes seems rather consistent.

**Table 2:** Average absolute number of cells (Av), standard deviation (*SD*), and coefficient of variation (CV), both hemispheres summed.

		Region							
		
		Anterior	Remaining	Olfactory	Hippo-	Diencephalon	Brainstem	Cerebellum	Whole
		neocortex	cortex	bulb	campus	plus Basal Ganglia			brain
		
	*N*	11	11	11	11	11	11	11	11
**Total cells**	Av	**2.70E + 06**	**1.65E + 07**	**1.08E + 07**	**3.69E + 06**	**1.09E + 07**	**1.21E + 07**	**3.85E + 07**	**9.52E + 07**
	*SD*	406782	2.39E + 06	2.35E + 06	457282	1.11E + 06	2.21E + 06	4.42E + 06	8.49E + 06
	CV	15.09%	14.51%	21.79%	12.38%	10.17%	18.19%	11.47%	8.91%
**Oligos**	Av	**569,986**	**4.41E + 06**	**1.01E + 06**	**887,014**	**3.69E + 06**	**3.68E + 06**	**3.13E + 06**	**1.74E + 07**
	*SD*	134,061	854,267	201,112	186,305	833,980	881,544	1,05E + 06	2,77E + 06
	CV	23.52%	19.39%	19.94%	21.00%	22.63%	23.98%	33.65%	15.98%
**Neurons**	Av	**541,442**	**4.98E + 06**	**5.65E + 06**	**829,351**	**3.01E + 06**	**3.39E + 06**	**3.20E + 07**	**5.04E + 07**
	*SD*	308,645	1.47E + 06	1.17E + 06	202,856	525,304	1.30E + 06	3.85E + 06	4.14E + 06
	CV	57.00%	29.55%	20.69%	24.46%	17.46%	38.44%	12.05%	8.23%
**Other cells**	Av	**1.58E + 06**	**7.10E + 06**	**4.11E + 06**	**1.98E + 06**	**4.25E + 06**	**5.07E + 06**	**3.42E + 06**	**2.75E + 07**
	*SD*	273,699	2.20E + 06	2.18E + 06	335,961	914,321	1.06E + 06	1.05E + 06	4.26E + 06
	CV	17.27%	31.06%	53.00%	16.99%	21.51%	20.90%	30.73%	15.49%


With the exception of olfactory bulb and cerebellum, which concentrate a great majority of neurons as compared with other cells, in every other region the proportion of oligodendrocytes tends to be similar to the proportion of neurons, varying between 20 and 40%. Interestingly, for the diencephalon plus basal ganglia, and for the brainstem, the three cell populations (neurons, oligodendrocytes, and other cells) seem to be more similarly distributed. Noticeably, in none of the analyzed regions oligodendrocytes outnumbered neurons and the other cells.

### Possible Allometric Scaling Across Brain Structures

In this section, we have posed and tested two hypotheses regarding brain scaling rules. The first hypothesis is that there may be a simple correlation between the mass of a structure and its number of neurons or oligodendrocytes (Herculano-Houzel et al., 2015). The second is that the numbers of oligodendrocytes and neurons may be positively correlated across brain structures. If present, these hypotheses would apply to animals of a particular lineage within a single species and might not reflect the scaling rules found across multiple species and orders.

**FIGURE 7:**
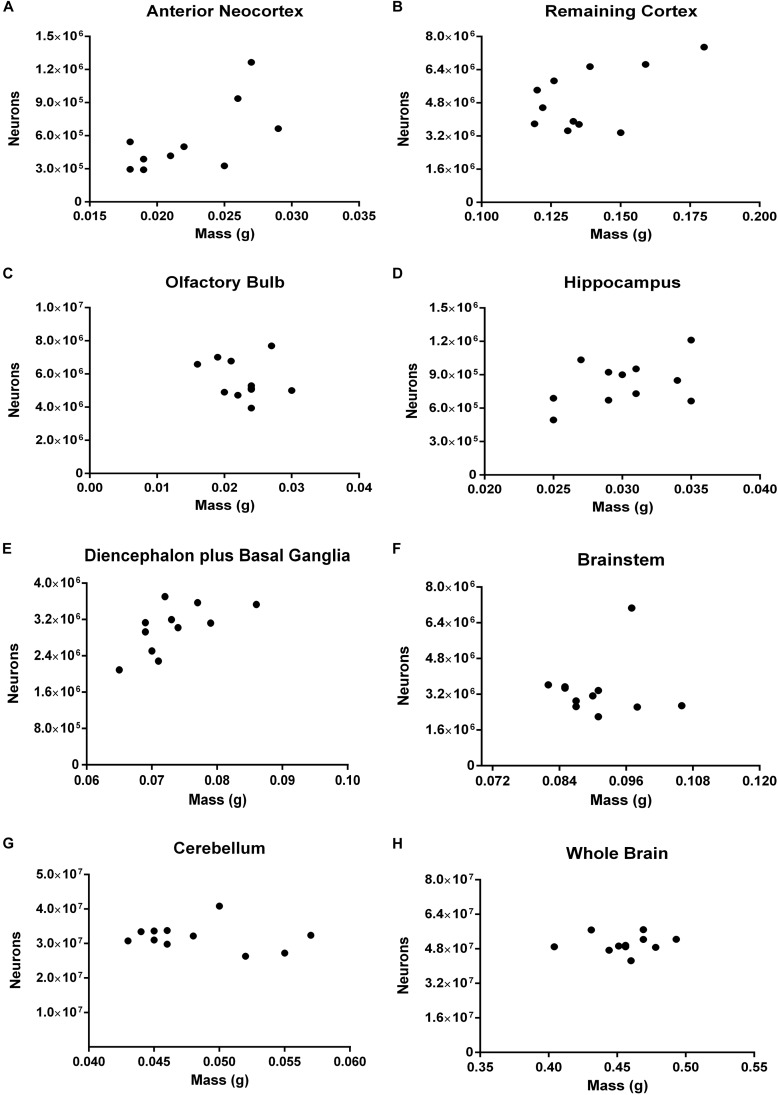
Spearman’s correlation between the absolute number of neurons and the mass of different ROIs. Dissected regions are shown from **(A–G)**. The data for the whole brain is displayed in **(H)**. **(A)** Anterior Neocortex: *rho* = 0.5812, *p* = 0.0649. **(B)** Remaining Cortex: *rho* = 0.3182, *p* = 0.3415. **(C)** Olfactory Bulb: *rho* = –0.1535, *p* = 0.6020. **(D)** Hippocampu**s**: *rho* = 0.2615, *p* = 0.4419. **(E)** Diencephalon plus Basal Ganglia: *rho* = 0.5968, *p* = 0.0569. **(F)** Brainstem: *rho* = –0.4439, *p* = 0.1628. **(G)** Cerebellum: *rho* = –0.1781, *p* = 0.5889. **(H)** Whole Brain: *rho* = 0.1963, *p* = 0.5603. Each dot represents one animal (*n* = 11).

Correlations between the number of neurons and the mass for most structures and for the whole brain have reached the threshold of statistical significance (Figure 7). Two ROIs showed moderate coefficients of correlation and borderline *p*-values: the anterior neocortex (*rho* = 0.5812, *p* = 0.0649) and the hippocampus (*rho* = 0.5505, *p* = 0.0863).

**FIGURE 8:**
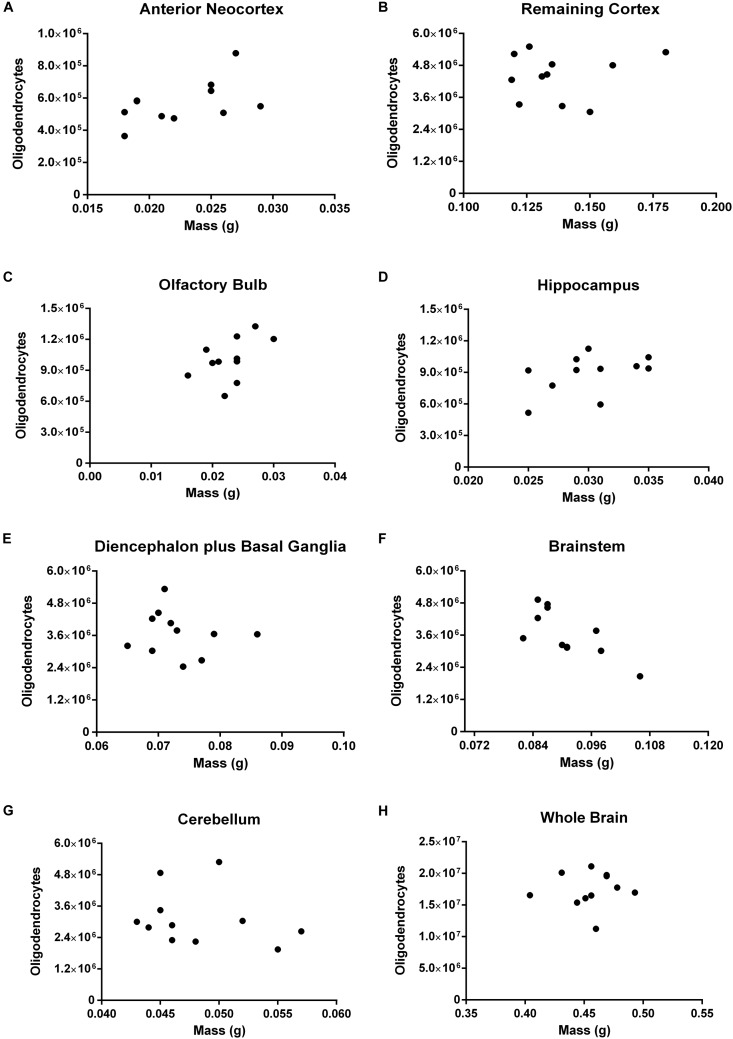
Spearman’s correlation between the absolute number of oligodendrocytes and mass. Dissected regions are shown from **(A–G)**. The data for the whole brain is displayed in **(H)**. **(A)** Anterior Neocortex: *rho* = 0.4302, *p* = 0.1868. **(B)** Remaining Cortex: *rho* = 0.0090, *p* = 0.9895. **(C)** Olfactory Bulb: *rho* = 0.5257, *p* = 0.0999. **(D)** Hippocampus: *rho* = 0.5505, *p* = 0.0863. **(E)** Diencephalon plus Basal Ganglia: *rho* = –0.2642, *p* = 0.4215. **(F)** Brainstem: *rho* = –0.7369, *p* = 0.0108. **(G)** Cerebellum: *rho* = –0.3196, *p* = 0.3283. **(H)** Whole Brain: *rho* = 0.1553, *p* = 0.6472. Each dot represents one animal (*n* = 11).

Concerning oligodendrocytes, we found a strong negative correlation between the number of these cells and the mass only for the brainstem (*rho* = -0.7369, *p* = 0.0108). In no other ROI nor the brain as a whole, the correlation has reached the threshold of statistical significance (Figure 8). For the olfactory bulb (*rho* = 0.5257, *p* = 0.0999) and the hippocampus (*rho* = 0.5505, *p* = 0.0863) the coefficients of correlation are moderate and the *p*-value close to significance.

**FIGURE 9:**
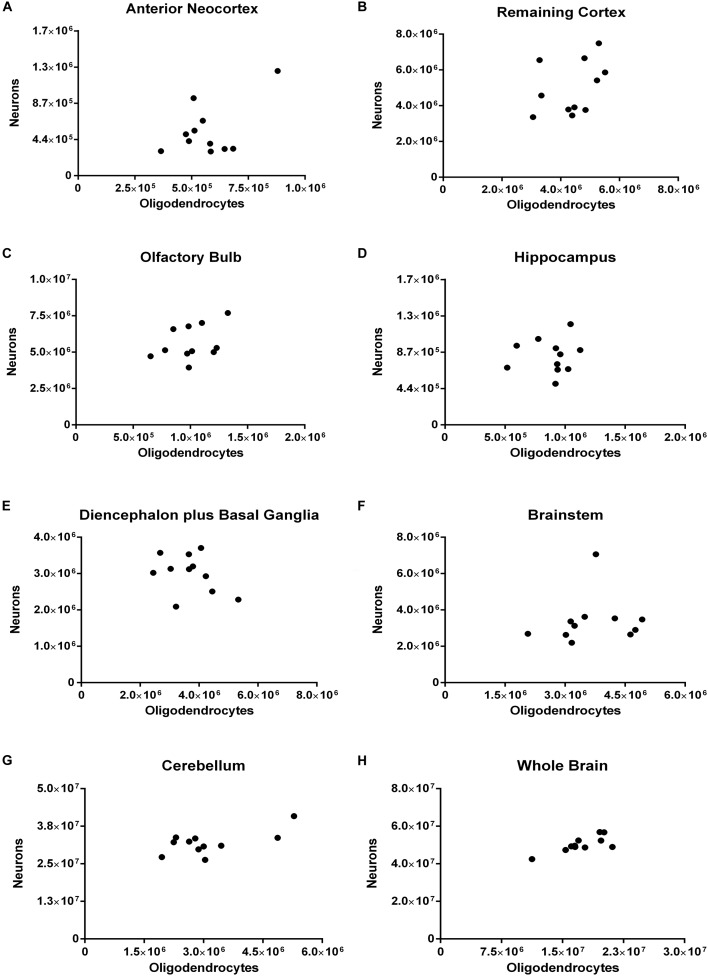
Spearman’s correlation between the absolute numbers of neurons and of oligodendrocytes. Dissected regions are shown from **(A–G)**. The data for the whole brain is displayed in **(H)**. **(A)** Anterior Neocortex: *rho* = 0.0272, *p* = 0.9462. **(B)** Remaining Cortex: *rho* = 0.4727, *p* = 0.1457. **(C)** Olfactory Bulb: *rho* = 0.4273, *p* = 0.1928. **(D)** Hippocampus: *rho* = 0.0545, *p* = 0.8812. **(E)** Diencephalon plus Basal Ganglia: *rho* = –0.3000, *p* = 0.3713. **(F)** Brainstem: *rho* = 0.3727, *p* = 0.2608. **(G)** Cerebellum: *rho* = 0.2364, *p* = 0.4854. **(H)** Whole Brain: *rho* = 0.5909, *p* = 0.0609. Each dot represents one animal (*n* = 11).

In none of the dissected regions there was any significant correlation between the number of oligodendrocytes and that of neurons. It is worth noticing that for the whole brain, however (*rho* = 0.5909, *p* = 0.0609), the coefficient of correlation was rather moderate, and the *p*-value, borderline (Figure 9).

## Discussion

### A Universal and Specific Nuclear (Pan-)Marker for Oligodendrocytes

The purpose of estimating with precision the absolute number of the different cell types in the nervous system, whichever the method used to sample the cells to count, requires a universal and specific marker. Such a (pan-)marker is defined as universal if it is capable of labeling all (or almost all) individual cells of a given type/subtype, and it is understood as specific if it can label only that particular type/subtype, and no other. These criteria have been largely fulfilled by NeuN, a widely used marker of neuronal nuclei, despite some known exceptions as the cerebellar Purkinje cells, mitral cells of the olfactory bulb, and inferior olive neurons (Mullen et al., 1992). In addition, to be usable with the isotropic fractionator, these markers must be nuclear, since that method requires cell membrane dissolution, with preservation of the nuclear membrane (Herculano-Houzel and Lent, 2005).

We here demonstrate that oligodendrocyte transcription factor 2 (Olig2) is a nuclear marker with the above characteristics, eligible to count absolute numbers of oligodendrocytes in the brain, using the isotropic fractionator. Even though Olig2 has already been used for oligodendrocyte quantification in tissue sections (Falkai et al., 2016; Liu et al., 2016), as well as with the isotropic fractionator (Hayashi et al., 2012; Chen et al., 2015), to our knowledge it has not been validated properly, and for this reason the total number of oligodendrocytes in the brain remains undetermined. Therefore, it was necessary to confirm that Olig2 is indeed expressed only by the oligodendrocytic lineage and in all its subtypes, namely: (1) gray matter oligodendrocytes apposed to neurons, called satellite or perineuronal (Szuchet et al., 2008); (2) those located in the white matter (myelinating cells in fasciculi and tracts); and (3) those related to brain blood vessels (perivascular oligodendrocytes), shown both in adult humans (Yokoo et al., 2004) and in developing animals, related to the migration of OPCs along blood vessels (Tsai et al., 2016).

In addition, it was relevant to confirm whether the absolute number of Olig2+ cells comprised only one stage of maturation (e.g., myelinating oligodendrocytes) or many stages, since several studies show its expression in oligodendrocyte progenitor cells (Dimou et al., 2008; Sim et al., 2011; Barateiro and Fernandes, 2014). We have systematically tested it in different dissectible regions of the C57BL/6 murine brain. Since oligodendrocytes are present in the spinal cord as well (Hall et al., 1996), it is conceivable that Olig2 also applies to this neural region, although the absolute number of cells therein remains to be determined.

Therefore, since Olig2 is expressed in all undifferentiated oligodendrocytes, when does it start to be expressed? In fact, it is known that Olig2 is expressed even sooner than detected by the well-known OPC markers such as the chondroitin sulfate proteoglycan NG2 (also known as *csp4*) and the alpha receptor for platelet-derived growth factor (PDGFRα) (Nishiyama et al., 1996), expressed in response to Sonic Hedgehog (Lu et al., 2000). This makes it the earliest marker of committed oligodendrocyte progenitor cells. Both NG2 and PDGFRα bind and form a complex that delivers PDGF efficiently to the receptor (Hart et al., 1989), thus characterizing an additional non-neuronal cell type in the CNS, known as oligodendrocyte progenitor cell, polydendrocyte, or oligodendrocyte type 2 astrocyte (O-2A progenitors), which comprises about 5% of the total cell population (Dawson et al., 2003). The OPCs are considered the most proliferative cells in the adult brain (Dawson et al., 2003). Among the PDGFRα+ cells, an average of 10% colocalizes with the cell cycle marker KI-67 (Simon et al., 2011; Marques et al., 2016). We here confirmed that counts of Olig2+ cells include these precursor cells, since it colocalizes with KI-67 and NG2 (Figures 4A,B).

Nonetheless, despite being considered a reliable marker for OPCs, NG2 is not completely specific, since we and others have identified NG2+ cells that do not colocalize with Olig2, probably because NG2 labels pericytes as well (Marques et al., 2016). On the other hand, stereological and FACS studies in animals have shown that in both developing and adult brains, at least 90% of NG2 cells are Olig2+ in different regions, in contrast to a minor population of 25% of Olig2+ that also labels for NG2 in adults (Ligon et al., 2006). Even for the PDGFRα, considered a more specific marker for OPC that also expresses Olig2 (Hall et al., 1996; Sim et al., 2011), a recent study demonstrates a particular PDGFRα population which is Sox10 negative (another transcription factor that begins its expression at the same time as Olig2), differently from OPCs related to blood vessels (Marques et al., 2016). NG2+ or PDGFRα+ cells, thus, require a second specific marker such as Olig2 or Sox10 to identify the population committed to an oligodendrocytic fate. Based on these several lines of evidence, we conclude that Olig2 labeling covers the whole population of oligodendroglial lineage cells (from young progenitors to the mature cells) and not only a single stage, as is the case of NeuN, which labels only differentiated neurons.

As the OPCs mature, they downregulate NG2 and PDGFRα, become fate-committed, and start expressing the oligodendrocyte marker O4 (Sommer and Schachner, 1981), which labels the pre-myelinating stage as well as G protein-coupled receptor 17 (GPR17) and CNPase. A full maturation is characterized by the expression of myelin-associated proteins such as myelin basic protein (MBP), myelin proteolipid protein (PLP), myelin-associated glycoprotein (MAG), and myelin oligodendrocyte glycoprotein (MOG) along with others such as CNPase (cell body and myelin) and the mature somatic marker CC1 (Figures 4C–E). Some studies have demonstrated that after brain injury (Tatsumi et al., 2008; Dimou and Götz, 2014) or *in vitro* manipulations (Raff et al., 1983; Behar et al., 1988), OPCs can generate astrocytes, sharing radial glia-like genes. This is why they are also called oligodendrocyte type 2 astrocytes. Besides, other authors have shown a colocalization between Olig2 and GFAP in postnatal (P7) animals (Marshall et al., 2005) and in neuronal progenitors during the embryonic period (E14) (Takebayashi et al., 2000). However, this issue has not been entirely clarified, since other studies have shown that Olig2 is limited to oligodendroglial cells (Wegner, 2001; Yokoo et al., 2004). We here tested whether some non-neuronal markers colocalized with Olig2, but we did not identify any one with this feature: astrocytes by use of ALDH1L-1, and neurons with NeuN (Figures 5A,D). Although there is emerging evidence of an Olig2-lineage of astrocytes (GFAP-negative) (Tatsumi et al., 2016, 2018), to date there is no robust quantitative analysis of this population. Furthermore, Olig2 did not co-label with basal lamina and endothelial cells markers such as Isolectin B4 and Collagen IV basement membrane. In addition, it does not colocalize with microglial IBA-1 marker (Figures 2B, 5B,C). However, as we have not assessed specificity in embryonic and neonatal animals in this work, we cannot assure whether the number of oligodendroglial cells during these developmental periods presents the same noise generated by other cells, such as astrocytes.

We concluded that Olig2 may be considered a universal and specific (pan-)marker for the oligodendrocytic lineage of cells in the adult mouse brain, being thus an appropriate nuclear proxy to determine the absolute number of these cells by use of the isotropic fractionator.

### The Absolute Number of Oligodendrocytes in the Mouse Brain

Having established that Olig2 is a nuclear (pan-)marker for oligodendrocytes, we sought to determine their absolute number in the mouse brain by use of the isotropic fractionator, and to challenge recent findings on intraspecific allometric relations between oligodendrocytes and mass, and between oligodendrocytes and neurons (Herculano-Houzel et al., 2015). Our first finding was that the adult C57BL/6 mouse brain, as a whole, contains around 17.4 million oligodendrocytes (∼20%), against 50.5 million neurons (∼50%) and 27.4 million other cells (∼30%). These numbers differ from previous work using Swiss mice (Herculano-Houzel et al., 2006), in which more neurons were reported in the whole brain (about 70 million) with the same methodology, potentially indicating a sizeable variation among different strains.

**FIGURE 10:**
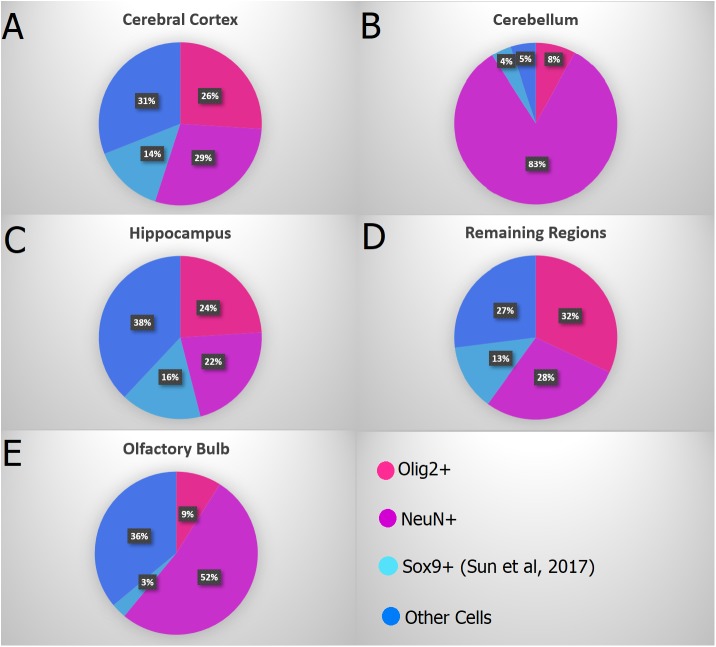
Combined data showing the proportion of Olig2+ nuclei in pink (oligodendrocytes), NeuN+ nuclei in purple (neurons), Sox9+ nuclei in light blue (astrocytes – Sun et al., 2017) and the remaining cells in dark blue in the main regions: **(A)** *Cerebral cortex* (*anterior neocortex* plus *remaining cortex*), **(B)** *Cerebellum*, **(C)** *Hippocampus*, **(D)** *Remaining Regions* (*Diencephalon* + *Basal Ganglia* + *Brainstem*), and **(E)** *Olfactory Bulb*. Both studies (Sun et al., 2017 and ours) used adult mice brains from the C57BL/6 lineage, allowing an integrated analysis of both data.

According to a recent study about the number of astrocytes in the murine brain using the isotropic fractionator (Sun et al., 2017), the number of these glial cells was shown not to surpass 20% of the total number of cells in any region (Figure 10). Taking together both data sets (Sun’s and ours), the percentage of non-neuronal, non-oligodendroglial and non-astroglial cells (=microglia + vascular + ependymal cells) would reach, roughly, 27–38% in most regions, though only 5% in the cerebellum.

Using stereology, Brasileiro-Filho et al. (1989) estimated the number of endothelial cells within the human cerebral cortex to be roughly twice the number of astrocytes. Coupling the estimates of neurons (NeuN+), oligodendrocytes (Olig2+), astrocytes (Sox9+) and extrapolating for the different regions of the murine brain according to the data provided by Brasileiro-Filho et al. (1989), we can examine the suggestion that oligodendrocytes compose 45–75% of glial cells (von Bartheld et al., 2016). Taking, as an example, our data in the murine cortex we arrive at roughly 19.2 million total cells, 4.9 million oligodendrocytes and 5.5 million neurons, and from the literature 14% of astrocytes (approximately 2.6 million: Sun et al., 2017) and 28% of endothelial cells (5.3 million: Brasileiro-Filho et al., 1989). By subtracting the number of neurons and endothelial cells from the total cell number, we end up with the number of glial cells (approximately 8.8 million).

These numbers lead to a proportion of 56% oligodendrocytes amongst all glia, allowing to conclude that, for the cortex, oligodendrocytes would comprise more than half the number of glial cells. Applying the same rationale for the other regions (and assuming that the proportion of endothelium and astrocytes remains roughly the same, 2:1) we arrive at the following percentages of oligodendrocytes/glial cells: 53% in the hippocampus, 72% in the remaining regions, 75% in the cerebellum and 23% in the olfactory bulb. Except for the olfactory bulb, then, in all regions oligodendrocytes comprise more than 50%, not surpassing 75%.

Another interesting observation, already noticed in other studies using the isotropic fractionator (Herculano-Houzel et al., 2006; Azevedo et al., 2009), and here confirmed, is that the brain is biased toward neurons mostly because of the cerebellum, a highly neuron-dense structure, making it the most numerous cell type in whole brain counts (Lent et al., 2012).

Although there is a growing body of evidence showing that the number of neurons positively correlates with brain mass across species, few studies have, so far, tackled this relation at an intraspecific level. Mass is determined by different factors, among them the cellularity of the brain. Since neurons comprise over 50% of the mouse brain, according to our data, and oligodendrocytes comprise around 20% of all cells, both of them certainly have a contribution to mass. Of course, besides cell numbers, other factors play a role in this case, such as the extracellular space, process arborization, degree of myelination, and vascularization.

We show here that, in C57BL/6 mice, the numbers of oligodendrocytes and neurons do not strongly correlate with mass in any of the analyzed structures. This may mean that while brain mass (and by consequence, neuronal numbers) may be important to assess functional complexity across species within the same order, it is of poor validity for the same aim among individuals of the same species/strains. Recent work has correlated mass and cortical folding with brain cellularity (Mota and Herculano-Houzel, 2015) by comparing species, but no similar attempt was made in the intraspecies level.

By relating neuronal with non-neuronal cells, strong positive correlations were previously described in the Swiss mouse across many regions of interest (Herculano-Houzel et al., 2015). We have tried to refine such findings by testing whether oligodendrocytes covary with neurons. Except for the whole brain (*rho* = 0.5909, *p* = 0.0609), however, we could not detect any moderate to strong correlation (on the verge of statistical significance) between the numbers of neurons and oligodendrocytes. Four possible explanations are: (1) the strong correlation observed between neuronal and non-neuronal cells may be due to astrocytes or other cell types, but not to oligodendrocytes; (2) differently from the Swiss mice, C57BL/6 is an isogenic lineage and its genetic variance is extremely low, tending to zero, thus if genetic variance is a necessary condition to determine correlation, we would not be able to detect it; (3) dissectible parts of the brain, as required for using the isotropic fractionator, may not reflect the subtle relations between oligodendrocytes and specific types of neurons (such as interneurons and long-projecting cells, for instance); (4) although similar techniques were employed, studies conducted in more than one laboratory may differ, causes varying from the use of reagents supplied by different companies, to different protocols. We have evidence (unpublished) that the estimated number of neurons in the Swiss mouse brain displays a larger variance when compared to C57BL/6, and also that the variation between different experimenters is rather small, not exceeding 3%.

Why is it of interest to look for a correlation between the number of neurons and that of oligodendrocytes? Since the numbers of the different cell types in the nervous system do not vary too much (see Figure 6), it is conceivable that a strict developmental program would regulate these numbers in each individual of the same species, possibly involving the reciprocal modulation of proliferation and cell death (Miller, 2002) when cell types start to emerge from precursors within the proliferative layers in the embryo. This assumption makes it also conceivable that numbers of neurons could be correlated with glial cell types, among them oligodendrocytes, what would mean that the general control of proliferation and death would be jointly regulated by species-specific developmental rules, resulting in correlated numbers. In favor of this hypothesis, stands the fact that oligodendrocytes share proliferating niches and migrating routes with neurons in the brain (e.g., the ventricular/subventricular zones, the ganglionic eminences) (van Tilborg et al., 2018). On the other hand, active communication between these two cell lineages (Maldonado and Angulo, 2015) may regulate myelination so that some cells would not require it (e.g., olfactory bulb interneurons, cerebellar granule cells), while some others would need it (e.g., long-projecting pyramids). The correlation hypothesis, although conceivable, did not prove to be true for large regions of the brains as those anatomically dissectible, according to our data. The relevance of this negative conclusion is that one should now search for independent mechanisms of number regulation of neural cell types along development, according to particular, more specific regions of the brain, or else by cell types. Focusing in these directions is now justified by previous exclusion of a more general, developmental mechanism of related regulation of cell numbers, as was our hypothesis.

Despite the absence of correlation in large, dissectible brain regions, it is still possible that specific subpopulations might be functionally related. As an example, Burne et al. (1996) provided evidence of a proportional relation between the number of axons and that of glial cells (microglia, oligodendrocytes, and astrocytes) in the mouse optic nerve. Although they do not use any statistical tool to model a possible correlation between these variables, they were able to show that a transgenic mouse expressing the human *bcl-2* gene, which had an increased number of axons, had also an increased number of glial cells when compared to the wild-type. Interestingly, transection of the optic nerves in rats was not able to impair migration of oligodendrocyte precursors and their differentiation into mature oligodendrocytes, whose densities were similar to those of the control rats (Ueda et al., 1999). The discovery of shared and/or interacting regulatory mechanisms between neurons and oligodendrocytes (Barres and Raff, 1999; Miller, 2002) supports the notion that, at least for specific cytoarchitectonic structures, the population of neurons and oligodendrocytes may be functionally connected and fluctuate together.

It is possible, therefore, that a positive correlation would be detected between neurons and oligodendrocytes if we take into consideration the myelin ensheathment in subpopulations of neurons of specific regions of the mouse brain, such as the cerebral cortex. Indeed, Micheva et al. (2016) have shown that half of neocortical myelin insulates axons of parvalbumin-positive basket interneurons. Since our analyzed regions of interest are composed by a mixture of different types of neurons (e.g., inhibitory and excitatory) and oligodendrocytes (premyelinating, myelinating, perineuronal, and perivascular), this diversity could explain the absence of correlation between neurons and oligodendrocytes, as seen in our work.

## Conclusion

We have shown that Olig2 transcription factor is a suitable marker for quantifying cells of the oligodendroglial lineage because: (1) it is nuclear, thus appropriate for the isotropic fractionator method; (2) it is specific, since it is not expressed in any nuclei other than those of oligodendrocytes; and (3) it is universal, since it labels all oligodendrocyte nuclei in the brain. Besides, since Olig2 is expressed early in development, counts comprise the proliferative and migratory, non-myelinating and myelinating cells. We have also shown that the adult C57BL/6 mouse brain contains on average: 95.2 million cells, of which 17.4 million are oligodendrocytes, about 18% of all cells, a number well below neurons (50.4 million) and other cells (27.5 million). In addition, we have provided not only the number of oligodendroglial cells per region, but also new evidence questioning a universal scaling relation between this cell type and other variables, such as the number of neurons and the mass of the analyzed structures.

## Author Contributions

BV-G, DG, DS, and RL designed the research. BV-G and DG performed experiments, analyzed the data, and wrote the manuscript. RL supervised the project and revised the manuscript.

## Conflict of Interest Statement

The authors declare that the research was conducted in the absence of any commercial or financial relationships that could be construed as a potential conflict of interest.
